# Warner’s Pocket Medical Dictionary of Today

**Published:** 1900-03

**Authors:** 


WARNER'S POCKET MEDICAL DICTIONARY OF TODAY. Comprising pronunciation and definition of 10,000 essential words and terms used in medicine and associated sciences, and tables of arteries, nerves, muscles, etc. Arranged for convenient reference. By WILLIAM R. WARNER. Copyright. Philadelphia: William R. Warner & Co. 1899.

Wm. R. Warner & Co., present the second edition of their pocket medical dictionary, a little volume of convenient size and of much practical value. Pronunciation, as well as the meaning of upwards of 10,000 medical terms is given, those very common words and phrases whose definition would but encumber the book without adding anything to the knowledge of the student, being omitted.

